# Inner diameters of the normal carotid arteries measured using three-dimensional digital subtraction catheter angiography: a retrospective analysis

**DOI:** 10.1186/s12883-021-02328-z

**Published:** 2021-07-26

**Authors:** Qingjing Tan, Chao Qin, Junwei Yang, Tianbao Wang, Haohai Lin, Cuiting Lin, Xiangren Chen

**Affiliations:** 1 Department of Neurology, First Affiliated Hospital of Guangxi University of Chinese Medicine, Nanning, Guangxi China; 2 Department of Neurology, First Affiliated Hospital of Guangxi Medical University, Nanning, Guangxi China

**Keywords:** Carotid arteries, Carotid bifurcation, Internal diameter, Digital subtraction angiography, Essen Stroke Risk Score

## Abstract

**Purpose:**

To obtain normal ranges for the inner diameters of the carotid arteries.

**Methods:**

This retrospective analysis included consecutive patients with disease-free carotid arteries who had undergone 3D-DSA at two hospitals in Nanning, Guangxi, between March 2013 and March 2018. Demographic and clinical characteristics, including Essen Stroke Risk Score (ESRS), were extracted from the medical records. The 3D-DSA data were used to calculate the inner diameters of the carotid arteries.

**Results:**

The analysis included 1182 patients (837 males) aged 58.81 ± 11.02 years. The inner diameters of the proximal carotid sinus (CS), CS bulge, distal CS, and common carotid artery (CCA) were larger on the right than on the left (*P* < 0.05). The inner diameters of the proximal CS, CS bulge, distal CS, and CCA on both sides were larger for males than females (*P* < 0.05). The inner diameters of the proximal CS, CS bulge, and distal CS on both sides were smaller for patients aged > 65 years than for patients aged ≤ 55 years (*P* < 0.05). Right CCA inner diameter did not vary with age, whereas left CCA inner diameter was larger for patients aged > 55 years than for patients aged ≤ 45 years (*P* < 0.05). The inner diameters of the proximal CS, CS bulge, and distal CS on both sides were smaller for patients with ESRS ≥ 3 than those with ESRS < 3 (*P* < 0.05).

**Conclusion:**

This study provides reference values for the internal diameters of normal carotid arteries. Carotid artery diameters varied with side, sex, and age.

**Supplementary Information:**

The online version contains supplementary material available at 10.1186/s12883-021-02328-z.

## Introduction

Stroke is one of the leading causes of death worldwide and a primary cause of disability in adults [1, 2]. Cerebrovascular accidents (CVAs) are a major cause of morbidity and mortality in China, where stroke incidence has been increasing [3]. The age-standardized incidence and prevalence of stroke in China are 1115/100,000 people and 247/100,000 person-years, respectively, and the mortality rate from CVAs is around 115/100,000 person-years [4]. Ischemic stroke accounts for 60–80% of all cases of CVA, with hemorrhagic stroke accounting for the remainder [2]. Atherosclerotic plaques in the carotid arteries are an important cause of ischemic stroke, accounting for more than 20% of cases [5]. The region of the carotid bifurcation is a common site for atherosclerotic plaques, with the majority of lesions located within the carotid bulb [6, 7].

**Fig. 1:**
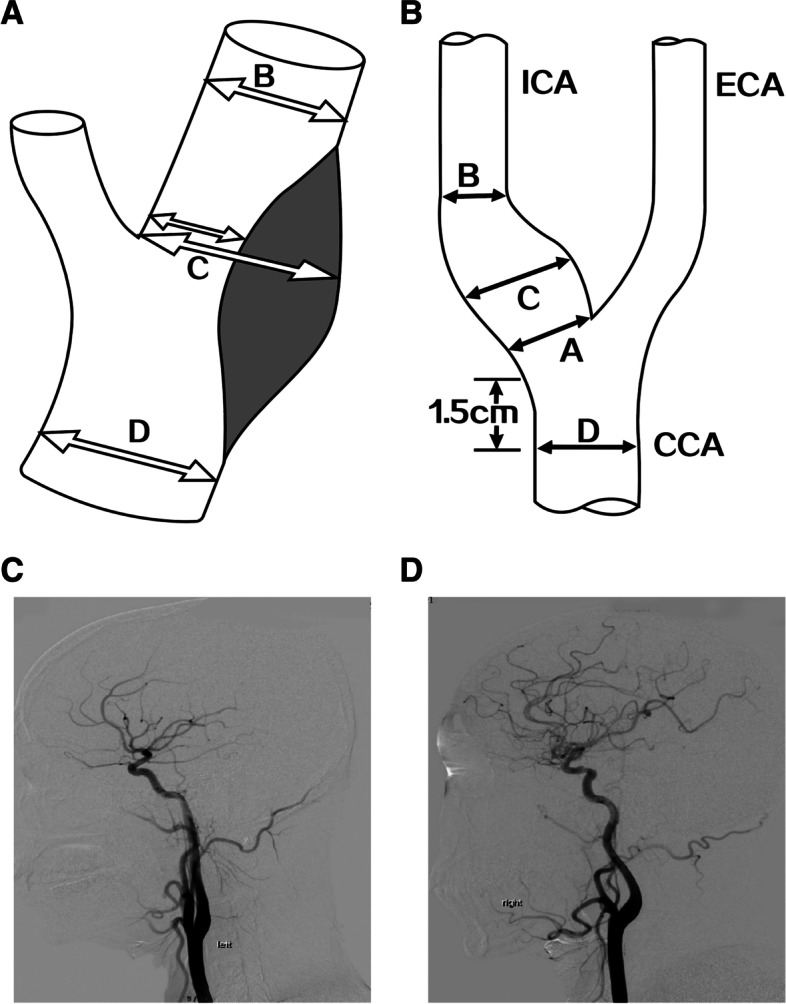
**A** Criteria for assessing the degree of carotid stenosis: NASCET criteria **B **Schematic diagram of the anatomical measurement part of carotid atery bifurcation **C**, **D** Anatomical structure of the carotid artery bifurcation displayed with three-dimensional (3D) DSA

Evaluation of the degree of carotid artery stenosis provides important information used to facilitate decision-making regarding the appropriate management strategy. At present, most assessments of the degree of carotid stenosis are performed using one of three criteria: the North American Symptomatic Carotid Endarterectomy Trial (NASCET) criteria [8, 9], the European Carotid Surgery Trial (ECST) criteria [10], and the common carotid (CC) method [11]. Although all three criteria rely on the measurement of the diameter of the residual lumen at the site of its maximal stenosis (measurement A in Fig. 1A), the three criteria differ with regard to the reference diameter used as the denominator: the NASCET method uses the normal distal internal carotid artery (ICA; measurement B in Fig. 1A), the ECST method uses the estimated width of the original artery at the site of maximal narrowing (measurement C in Fig. 1A), and the CC method uses the proximal common carotid artery (CCA; measurement D in Fig. 1A) [12]. In view of the likelihood of inter-individual variation (e.g., between people of difference ages or sexes) as well as intra-individual variation (between left and right sides), detailed knowledge of the normal ranges for carotid artery diameter at these different locations might help to refine the cut-off values used in the clinical diagnosis of carotid stenosis.

Various techniques have been utilized to evaluate the degree of carotid artery stenosis, including computed tomography angiography (CTA), contrast-enhanced magnetic resonance angiography (MRA), and Doppler ultrasonography [13–16]. Although all three of the above noninvasive techniques have their individual merits, it is widely acknowledged that their accuracy is limited and inferior to that of digital subtraction catheter angiography (DSA), which is currently considered the ‘gold-standard’ method for assessing the extent of carotid artery stenosis. Despite this, to our knowledge, no previous studies have used DSA to obtain reference values for the normal (i.e., disease-free) carotid artery. Therefore, the aim of this retrospective analysis was to obtain normal ranges for the diameter of the carotid artery at four locations (see Fig. 1B–D) using three-dimensional (3D) DSA. It was anticipated that the data would provide basic anatomic values for use in medical applications and clinical research in the field of carotid artery stenosis.

## Patients and methods

### Study design and study participants

This retrospective analysis included consecutive patients admitted to the Department of Encephalopathy, First Affiliated Hospital of Guangxi University of Traditional Chinese Medicine (Nanning, Guangxi, China), and the Department of Neurology, First Affiliated Hospital of Guangxi Medical University (Nanning, Guangxi, China) between March 2013 and March 2018. The inclusion criteria were as follows: (1) 3D-DSA of the whole brain was performed during hospitalization, and a complete 3D-DSA dataset was available; (2) the carotid artery was well-developed, and its structure could be clearly distinguished; (3) the 3D-DSA arterial phase scan of carotid bifurcation-related blood vessels included the distal CCA, carotid sinus (CS) and origins of the ICA and external carotid artery (ECA); (4) no evidence of stenotic lesions in carotid bifurcation-related blood vessels; (5) blood lipids, serum homocysteine (Hcy) and blood glucose levels were measured during hospitalization; and (6) Essen Stroke Risk Score (ESRS) had been determined. The ESRS is an easy-to-use 9-point scale that has been validated as a tool for risk stratification of patients with regard to recurrent stroke and combined cardiovascular events [17–19]. The exclusion criteria were: (1) DSA showed clear vascular stenosis (including congenital or acquired stenosis or obstruction) at the carotid bifurcation; (2) DSA demonstrated cervical hemangiectasis or carotid aneurysms; (3) head and neck bone deformities; (4) presence of obvious vessel compression by soft tissue masses; (5) severe heart, liver or renal insufficiency resulting in intolerance of the DSA examination; (6) pulmonary dysfunction; (7) malignancy; (8) tuberculosis or severe systemic infection; (9) allergy to iodine contrast agents; and (10) data required for the analysis were missing. This study was approved by the ethics committees of the First Affiliated Hospital of Guangxi University of Traditional Chinese Medicine and the First Affiliated Hospital of Guangxi Medical University, and the requirement for informed consent was waived because the analysis was retrospective and anonymized.

### Whole-brain 3D-DSA

3D-DSA was performed using an Axiom Artis dBC system (Siemens AG, Munich, Germany) or a Discovery IGS 730 system (GE Healthcare, Chicago, IL, USA). Angiography was developed from the aortic arch to clearly show the vascular structures of the carotid bifurcation (the distal CCA, CS, and initial segment of the ICA). The patient was placed in the supine position, and the right groin area was disinfected and isolated using drapes. The right femoral artery was punctured with the Seldinger technique, and the aortic arch was intubated. A pigtail catheter was used for supra-aortic angiography and whole-brain perfusion imaging. A non-ionic contrast agent (300 mg/L Ioproline 370 injected at a pressure of 300 PSI) was administered during angiography, and the carotid arteries were images in three views: anteroposterior, lateral, and oblique. The original 3D-DSA image was analyzed on a PACS system. The morphology and structures of the bilateral carotid bifurcations were evaluated from 2 cm above the initiation of the ICA to 1.5 cm below the level of the carotid bifurcation. A measurement tool provided in the software (Antai Technology Co. Ltd., Dongguan, Guangdong, China) was used to measure the inner diameter of the ICA at its proximal part (measurement A in Fig. 1B), the bulge of the ICA (measurement C in Fig. 1B), the ICA at its distal part (measurement B in Fig. 1B), and the CCA at approximately 1.5 cm below the level of the carotid bifurcation (measurement D in Fig. 1B). Measurements were made for both the left and right sides. To minimize measurement errors, three experienced radiologists conducted six successive measurements each (to reduce sampling errors), and the average value was used for the analysis.

### Collection of demographic and clinical data

The following clinical information was extracted from the medical records: age; sex; presenting symptoms and signs (e.g., focal neurological deficits such as hemiplegia, aphasia, sensory disorders and ataxia); results of blood tests, including fasting blood glucose, postprandial blood glucose, glycated hemoglobin, total cholesterol (TC), triglycerides (TG), high-density lipoprotein (HDL), low-density lipoprotein (LDL), and serum homocysteine (Hcy); family history; smoking history; alcohol consumption; history of hypertension (confirmed previous diagnosis or taking antihypertensive drugs); history of diabetes mellitus (confirmed previous diagnosis or taking hypoglycemic drugs); history of dyslipidemia, defined as TC > 5.2 mmol/L, TG > 2.3 mmol/L, LDL > 2.6 mmol/L, HDL ≤ 1.0 mmol/L, or taking lipid-lowering drugs; history of atrial fibrillation, coronary heart disease and/or other heart disease; history of previous cerebral infarction, transient ischemic attack, myocardial infarction or peripheral arterial disease; any other relevant medical history; and ESRS. The ESRS was used to categorize patients into a low-risk group (< 3 points) and a high-risk group (≥ 3 points) [17, 20, 21].

### Statistical analysis

The analysis was performed using SPSS 16.0 (SPSS Inc., Chicago, IL, USA). Quantitative data are described as the mean ± standard deviation, and qualitative data are expressed as *n* (%). Comparisons between the two groups were made using the t-test for independent samples. Comparisons between multiple groups were made using the F-test (one-way analysis of variance). The correlation between two variables was analyzed using Pearson correlation analysis. *P* < 0.05 was taken to indicate a significant difference.

## Results

### Baseline characteristics of the study participants

A total of 1394 patients who underwent 3D-DSA examination were screened for eligibility, and 212 were excluded due to DSA showed clear vascular stenosis at the carotid bifurcation (n = 138), DSA demonstrated cervical hemangiectasis or carotid aneurysms (n = 31), and head and neck bone deformities or presence of obvious vessel compression by soft tissue masses (n = 43). A total of 1182 hospitalized patients (837 males, 70.81%) who met the selection criteria were included in the analysis. The participants were aged 58.81 ± 11.02 years (range, 28–85 years). The ethnicity of the patients was Han (n = 653, 55.25%), Zhuang (n = 352, 29.78%), or others (n = 177, 14.97%). The clinical characteristics of the study participants are presented in Table 1. Most patients were hospitalized because of dizziness or intracranial vascular disease with cerebral infarction or cerebral hemorrhage. The reason for performing whole-brain 3D-DSA in these patients was the identification by CTA of cerebrovascular abnormalities (such as cerebral hemangiomas or cerebral arteriovenous malformations) or cerebrovascular stenosis/occlusion that needed further investigation. Among the 1182 patients, there were 969 (81.98%) with ischemic cerebrovascular diseases, including 610 (51.61%) with cerebral infarction, 155 (13.11%) with transient ischemic attack, and 204 (17.26%) with posterior circulation ischemia, and 213 (18.02%) with cerebral hemorrhage.

**Table 1:** Baseline clinical characteristics of the 1182 study participants included in the analysis

Characteristic	Value
Age (years), mean ± standard deviation	58.81 ± 11.02
Age range (years)	28–85
Age < 65 years, *n* (%)	816 (69.04%)
Age 65–75 years, *n* (%)	297 (25.13%)
Age > 75 years, *n* (%)	69 (5.84%)
Male, *n* (%)	837 (70.81%)
Ethnicity	
Han	653 (55.25%)
Zhuang	352 (29.78%)
Others	177 (14.97%)
History of smoking, *n* (%)	507 (42.89%)
History of alcohol consumption, *n* (%)	312 (26.40%)
Hypertension, *n* (%)	879 (74.37%)
Diabetes mellitus, *n* (%)	315 (26.65%)
Previous myocardial infarction, *n* (%)	30 (2.54%)
Previous other heart diseases^a^, *n* (%)	192 (16.24%)
Previous peripheral vascular disease, *n* (%)	9 (0.76%)
Previous history of transient ischemic attack or ischemic stroke, *n* (%)	261 (22.08%)
Dyslipidemia, *n* (%)	714 (60.41%)
Hyperhomocysteinemia, *n* (%)	828 (70.05%)
Diagnosis	
Ischemic cerebrovascular diseases	969 (81.98%)
Cerebral infarction	610 (51.61%)
Transient ischemic attack	155 (13.11%)
Posterior circulation ischemia	204 (17.26%)
Cerebral hemorrhage	213 (18.02%)

^a^ Excluding cardiac infarction and atrial fibrillation

### Comparisons of the inner diameters of the left and right carotid arteries

Table 2 compares the inner diameters of the carotid arteries between the left and right sides. Notably, the inner diameters of the proximal CS, CS bulge, distal CS, and CCA were all significantly larger on the right side than on the left side (*P* < 0.05 for all; Table 2). The largest absolute difference was observed for the CCA, with the mean diameter of the right CCA being 0.65 mm greater than that of the left CCA.

**Table 2:** Comparisons of the inner diameters of the left and right carotid arteries

Inner diameter measured	Range (95% confidence interval)		Mean ± standard deviation		*P*
	Left (*n* = 1182)	Right (*n* = 1182)	Left (*n* = 1182)	Right (*n* = 1182)	
Proximal carotid sinus (mm)	6.53–15.11 (10.55–11.09)	7.06–15.60 (11.06–11.60)	10.82 ± 2.19	11.33 ± 2.18	0.009
Carotid sinus bulge (mm)	7.01–14.97 (10.74–11.24)	7.39–15.35 (11.12–11.62)	10.99 ± 2.03	11.37 ± 2.03	0.038
Distal carotid sinus (mm)	5.50–10.12 (7.73–8.01)	5.83–10.37 (7.97–8.25)	7.81 ± 1.18	8.10 ± 1.16	0.020
Common carotid artery (mm)	8.24–13.46 (10.69–11.01)	8.85–14.15 (11.34–11.66)	10.85 ± 1.33	11.50 ± 1.35	< 0.001

### Comparisons of the inner diameters of the carotid arteries between males and females

The inner diameters of the carotid arteries are compared between males and females in Table 3. The inner diameters of the proximal CS, CS bulge, distal CS, and CCA were significantly larger for males than for females on both the left and right sides (*P* < 0.05 for all; Table 3).

**Table 3:** Comparisons of the inner diameters of the carotid arteries between males and females

Inner diameter measured	Range (95% confidence interval)		Mean ± standard deviation		*P*
	Male (*n* = 837)	Female (*n* = 345)	Male (*n* = 837)	Female (*n* = 345)	
Left proximal carotid sinus (mm)	6.55–15. 41 (10.66–11.30)	6.65–13.99 (9.836–10.80)	10.83 ± 2.32	10.13 ± 1.96	0.030
Right proximal carotid sinus (mm)	6.92–15.86 (11.24–11.88)	6.96–13.98 (10.17–11.06)	11.35 ± 2.28	10.37 ± 1.79	< 0.001
Left carotid sinus bulge (mm)	6.96–15.16 (10.85–11.44)	6.94–13.84 (10.04–10.95)	10.96 ± 2.23	10.32 ± 1.89	0.044
Right carotid sinus bulge (mm)	7.13–15.59 (11.26–11.86)	7.17–14.11 (10.34–11.23)	11.29 ± 2.18	10.54 ± 1.80	0.009
Left distal carotid sinus (mm)	5.25–11.21 (7.81–8.15)	5.27–10.17 (7.28–7.82)	7.98 ± 1.19	7.54 ± 1.08	0.010
Right distal carotid sinus (mm)	5.86–10.44 (8.06–8.38)	5.69–10.01 (7.47–7.98)	8.48 ± 1.45	7.87 ± 1.09	0.001
Left common carotid artery (mm)	7.74–13.82 (10.85–11.20)	7.36–13.00 (9.94–10.63)	11.03 ± 1.28	10.25 ± 1.39	< 0.001
Right common carotid artery (mm)	8.94–14.32 (11.52–11.88)	8.00–13.34 (10.56–11.13)	11.65 ± 1.37	10.71 ± 1.23	< 0.001

### Comparisons of the inner diameters of the carotid arteries between different age groups

Table 4 illustrates that there was a clear trend for the inner diameters of the proximal CS, CS bulge, and distal CS on both the left and right sides to decrease with increasing age. Notably, the inner diameters of the proximal CS, CS bulge, and distal CS on both sides were significantly smaller for patients aged > 65 years than for patients aged ≤ 45 years or 46–55 years (*P* < 0.05 for all; Table 4). The right CCA showed no obvious change in inner diameter with increasing age, although the inner diameter of the left CCA was significantly larger for patients aged 55–65 years or > 65 years than for patients aged ≤ 45 years (*P* < 0.05; Table 4).

**Table 4:** Comparisons of the inner diameters of the carotid arteries between different age groups

Inner diameter measured	Range (95% confidence interval)				Mean ± standard deviation				*P*
	≤ 45 years (*n* = 231)	46–55 years (*n* = 276)	56–65 years (*n* = 375)	> 65 years (*n* = 300)	≤ 45 years (*n* = 231)	46–55 years (*n* = 276)	56–65 years (*n* = 375)	> 65 years (*n* = 300)	
Left proximal carotid sinus (mm)	8.44–14.72 (11.04–12.13)	7.50–15.45 (10.85–11.89)	6.33–15.35 (10.35–11.34)	5.89–14.01 (9.47–10.43)	11.58 ± 1.59	11.37 ± 2.08	10.84 ± 2.30	9.95 ± 2.07^a,b,c^	< 0.001
Right proximal carotid sinus (mm)	8.71–15.13 (11.36–12.47)	7.46–16.24 (11.28–12.42)	6.81–15.79 (10.82–11.78)	6.69–14.61 (10.18–11.11)	11.92 ± 1.64	11.85 ± 2.24	11.30 ± 2.29	10.65 ± 2.02^a,b,c^	0.003
Left carotid sinus bulge (mm)	8.43–14.51 (10.94–11.99)	7.69–15.13 (10.94–11.88)	6.69–15.00 (10.39–11.31)	6.43–14.71 (10.08–11.06)	11.47 ± 1.55	11.41 ± 1.90	10.85 ± 2.12	10.57 ± 2.11^a,b^	0.036
Right carotid sinus bulge (mm)	8.26–15.74 (11.35–12.64)	7.55–15.71 (11.11–12.16)	7.27–15.35 (10.88–11.75)	7.14–14.48 (10.37–11.25)	12.00 ± 1.91	11.63 ± 2.08	11.31 ± 2.06	10.81 ± 1.87^a,b^	0.017
Left distal carotid sinus (mm)	6.38–10.74 (8.19–8.94)	6.45–10.25 (8.11–8.58)	5.42–9.78 (7.24–7.74)	5.14–9.84 (7.35–7.86)	8.56 ± 1.11	8.35 ± 0.97	7.60 ± 1.11^a,b^	7.49 ± 1.19^a,b^	< 0.001
Right distal carotid sinus (mm)	6.87–10.95 (8.56–9.26)	6.59–10.39 (8.25–8.73)	5.53–9.99 (7.53–7.99)	5.83–9.75 (7.57–8.02)	8.91 ± 1.04	8.49 ± 0.97	7.79 ± 1.00^a,b^	7.76 ± 1.14^a,b^	< 0.001
Left common carotid artery (mm)	8.22–12.54 (10.01–10.76)	8.22–13.56 (10.56–11.22)	8.15–13.71 (10.72–11.28)	8.53–13.47 (10.71–11.03)	10.38 ± 1.10	10.89 ± 1.36	10.93 ± 1.42^a^	11.00 ± 1.26^a^	0.122
Right common carotid artery (mm)	9.06–13.34 (10.83–11.57)	8.72–14.40 (11.20–11.91)	8.66–14.46 (11.26–11.86)	9.23–13.90 (11.29–11.82)	11.20 ± 1.09	11.56 ± 1.45	11.56 ± 1.48	11.56 ± 1.19	0.529

^a^ *P* < 0.05 vs. ≤ 45 years group; ^b^ *P* < 0.05 vs. 46–55 years group; ^c^ *P* < 0.05 vs. 56–65 years group

Pearson correlation analysis was performed to further characterize the associations between the various carotid artery diameters and age. As detailed in Table 5, inner diameter was significantly negatively correlated with age for the proximal CS, CS bulge, and distal CS on both sides (*P* < 0.05 for all). Left CCA inner diameter was weakly positively correlated with age (*P* < 0.05), whereas right CCA inner diameter showed no significant correlation with age (Table 5).

**Table 5:** Pearson correlation analysis of the relation between the inner diameters of the carotid arteries and patient age

Inner diameter measured	Pearson correlation coefficient (*r*)	*P*
Left proximal carotid sinus (mm)	-0.256	< 0.001
Right proximal carotid sinus (mm)	-0.204	0.001
Left carotid sinus bulge (mm)	-0.158	0.011
Right carotid sinus bulge (mm)	-0.204	0.001
Left distal carotid sinus (mm)	-0.284	< 0.001
Right distal carotid sinus (mm)	-0.335	< 0.001
Left common carotid artery (mm)	0.127	0.035
Right common carotid artery (mm)	0.062	0.306

### Comparisons of the inner diameters of the carotid arteries between groups stratified according to ESRS

The inner diameters of the bilateral proximal CS, CS bulge, and distal CS were significantly smaller for patients with an ESRS ≥ 3 points than for patients with an ESRS < 3 points (*P* < 0.05 for all; Table 6). However, the inner diameters of the left and right CCA did not differ significantly between patients with an ESRS ≥ 3 points and those with an ESRS < 3 points (Table 6).

**Table 6:** Comparisons of the inner diameters of the carotid arteries between groups stratified according to Essen Stroke Risk Score

Inner diameter measured	Range (95% confidence interval)		Mean ± standard deviation		*P*
	ESRS < 3 (*n* = 678)	ESRS ≥ 3 (*n* = 504)	ESRS < 3 (*n* = 678)	ESRS ≥ 3 (*n* = 504)	
Left proximal carotid sinus (mm)	6.79–15.41 (10.80–11.48)	5.93–14.13 (9.84–10.69)	11.14 ± 2.22	10.26 ± 2.02	0.002
Right proximal carotid sinus (mm)	7.17–15.83 (11.16–11.84)	6.68–15.00 (10.61–11.49)	11.49 ± 2.21	11.01 ± 2.08	0.001
Left carotid sinus bulge (mm)	7.27–14.95 (10.80–11.48)	5.74–14.92 (10.16–11.08)	11.08 ± 2.01	10.33 ± 2.34	0.007
Right carotid sinus bulge (mm)	7.29–15.33 (11.25–11.86)	6.57–15.07 (10.61–11.52)	11.55 ± 1.95	11.06 ± 2.12	< 0.001
Left distal carotid sinus (mm)	5.38–10.82 (7.85–8.20)	5.30–9.920 (7.37–7.85)	8.02 ± 1.16	7.61 ± 1.18	0.006
Right distal carotid sinus (mm)	5.94–10.92 (8.11–8.44)	5.00–11.58 (7.58–8.05)	8.27 ± 1.13	7.82 ± 1.14	0.002
Left common carotid artery (mm)	8.13–13.47 (10.53–10.94)	8.45–11.02 (10.80–11.30)	10.73 ± 1.36	11.05 ± 1.28	0.059
Right common carotid artery (mm)	8.86–14.08 (11.27–11.67)	8.56–14.32 (11.30–11.85)	11.47 ± 1.33	11.55 ± 1.38	0.083

*ESRS* Essen Stroke Risk Score

## Discussion

This study used 3D-DSA, which is considered a gold-standard technique for the evaluation of carotid artery stenosis, to measure the inner diameters of the carotid arteries at the carotid bifurcation. A notable finding was that the inner diameters of the proximal CS, CS bulge, distal CS, and CCA were larger on the right side than on the left side. Furthermore, the inner diameters of the proximal CS, CS bulge, distal CS, and CCA on both sides were larger in males than in females. Additionally, the inner diameters of the proximal CS, CS bulge, and distal CS on both sides were smaller for patients aged > 65 years than for patients aged ≤ 55 years, whereas left CCA inner diameter was larger for older patients than for younger patients. Interestingly, the inner diameters of the proximal CS, CS bulge, and distal CS on both sides were smaller for patients with ESRS ≥ 3 points than for patients with ESRS < 3 points. To the best of our knowledge, this is the first study to provide reference values for the internal diameters of the normal carotid arteries at the carotid bifurcation using 3D-DSA. Notably, the carotid artery diameters were found to vary with side, sex, and age. We anticipate that these data will prove useful for medical applications and clinical research in the field of carotid artery stenosis.

The present study used 3D-DSA to obtain the range, 95% confidence interval, and mean values for the inner diameters of the proximal CS, CS bulge, distal CS, and CCA. The values obtained in this analysis (see Table 2) were generally smaller than those reported by previous studies using CTA [22, 23], ultrasonography [24–27], and MRA [28]. There are several possible reasons for the apparent discrepancy between our findings and those of previous investigations. First, the spatial resolution of CTA, MRA, and ultrasonography are much lower than those of DSA [13]; hence the measurements are more susceptible to the effects of operator experience, patient cooperation, hemodynamic effects, and anatomic variation. Furthermore, the above methods are inferior to DSA for displaying small vessels, which can lead to an overestimation of the degree of stenosis. Second, CTA is sometimes associated with image distortion during reconstruction, which can affect the precision of the measurements. Third, the time-dependence of CTA (i.e., the timing of image acquisition after the administration of contrast) adds another layer of complexity to the measurement of lumen diameter, which can also introduce measurement errors. Fourth, the sample sizes of many of the above studies were small, which increases the likelihood of sampling errors.

We found that the inner diameters of the bilateral CS and distal CCA were greater in males than females, consistent with observations reported by several prior investigations using CTA, ultrasonography, or MRA [22, 24, 26–29]. One possible reason for the larger vessel diameters in males than females is that men have, on average, a larger body size. Nevertheless, it has been reported that women have smaller carotid arteries than men even after adjustment for body size [27]. Thus, other as yet unidentified factors may contribute to the observed differences in the inner diameters of the carotid arteries between males and females.

An interesting observation of this analysis was that the inner diameters of the CS and distal CCA were larger on the right side than on the left side. Several published studies have also reported a larger luminal diameter for the right CCA than for the left CCA [24, 27], although not all authors agree [22, 28, 29]. In view of the clear variation between left and right sides in this study, which included a large number of participants and used a technique with a high spatial resolution (3D-DSA), the failure of some other studies to detect such differences may be related to smaller sample size (which would reduce the power of the analysis) and use of methods with poorer spatial resolution. Possible reasons for the differences between left and right sides include differences between the structures, hemodynamics, and shear stresses of the carotid arteries. The left CCA originates directly from the aortic arch in the thorax, while the right CCA originates from the brachiocephalic trunk in the neck. Since the left CCA receives blood from the heart more directly, the larger blood flow impulse might affect its intima to a greater extent, promoting atherosclerosis and an increase in intima-media thickness (IMT) [23]. Nevertheless, this suggestion is speculative, and further research is needed to establish the mechanisms underlying the differences in carotid artery luminal diameter between the left and right sides.

We also found that the inner diameter of the bilateral CS decreased with increasing age, and this was particularly evident for patients who were > 65 years old, who had significantly smaller CS diameters than younger patients. The carotid bulb is by far the most common site of atherosclerotic plaque formation [6, 7], and since IMT has been shown to increase with age even after adjustment for cardiovascular risk factors [30], the progression of atherosclerosis in the CS may, at least in part, explain the effects of older age on CS diameters. By contrast, the CCA exhibited no association (right side) or a positive association (left side) with increasing age, similar to findings reported by other researchers [26, 27, 31]. Atherosclerotic plaque formation may be less pronounced in the distal CCA than in the carotid bulb because of less turbulent blood flow, and this may limit the effects of aging on IMT and CCA diameter.

The inner diameters of the bilateral CS were significantly smaller for patients with an ESRS ≥ 3 points than for patients with an ESRS < 3 points. Because ESRS is essentially an integration of age and other risk factors for atherosclerosis, an increase in ESRS would be predicted to be associated with an increase in the IMT and hence a decrease in the internal diameter of the CS.

Notably, different from the NASCET method, we measured the diameter of distal carotid sinus rather than distal ICA, and this study is also based on the model Y-shaped Average Human carotid Bifurcation, Y-AHCB [32, 33]. We agree that the method used in this study is not perfect, but it is quite commonly used in China [34–36]. In addition, the NASCET method has its limitations [37]. Especially, NASCET underestimates the degree of stenosis [38–40], and the relation between NASCET and other systems, such as ECST, is not linear [41].

This study has some limitations. Since this was a retrospective analysis, it is possible that the results are prone to selection bias or information bias. Because patients were enrolled from only two centers, the generalizability of the findings is not known. Furthermore, as the participants were recruited from encephalopathy and neurology departments, they were likely to be at higher vascular risk than the general population. No firm conclusions can be drawn regarding causality due to the cross-sectional design of the study. It should also be noted that although 3D-DSA can accurately reproduce the morphology of cerebral blood vessels, allowing accurate measurements of arterial luminal diameters, the technique is invasive, expensive, time-consuming, requires anesthesia, and may produce allergic reactions. Since this was a retrospective study, we were unable to obtain three-dimensional length information and then use the maximum diameter. In order to minimize measurement errors, three experienced radiologists tried their best to select the site with the maximum diameter and conducted six successive measurements each, and the average value was used for the analysis. This might affect the accuracy of measurement results to some extent. Finally, interindividual anatomical variations in the Circle of Willis [42] and other structures might influence the results, but such variations cannot be avoided. Therefore, when examining whether one patient has carotid artery stenosis, in addition to the inner diameters of the carotid arteries, we should also combine the clinical symptoms, signs, and other examination results.

In summary, this study is the first to obtain reference values for the internal diameters of the normal carotid arteries at the carotid bifurcation using 3D-DSA, a ‘gold-standard’ technique that has higher spatial resolution than CTA, MRA, and ultrasonography. Furthermore, the carotid artery diameters were found to vary between left and right sides and with sex and age. We anticipate that these data will prove useful for medical applications and clinical research in the field of carotid artery stenosis.

## Supplementary Information

**Additional file 1.**

## Data Availability

The data used and analyzed during the current study are available from the corresponding author on reasonable request.

## Abbreviations

CC: Common carotid
CCA: Common carotid artery
CS: Carotid sinus
CTA: Computed tomography angiography
CVAs: Cerebrovascular accidents
DSA: Digital subtraction catheter angiography
ECA: External carotid artery
ECST: The European Carotid SurgeryTrial
ESRS: Essen Stroke Risk Score
Hcy: Homocysteine
ICA: Internal carotid artery
IMT: Intima-media thickness
MRA: Magnetic resonance angiography
NASCET: The North American Symptomatic Carotid Endarterectomy Trial
TC: Total cholesterol
TG: Triglycerides
HDL: High-density lipoprotein
LDL: Low-density lipoprotein
3D: Three-dimensional
