# Supplementary material for: Inner diameters of the normal carotid arteries measured using three-dimensional digital subtraction catheter angiography: a retrospective analysis
Source: BMC Neurol. 2021 Jul 26;21:292. doi: 10.1186/s12883-021-02328-z (PMC8311942; doi:10.1186/s12883-021-02328-z)
Supplement: Supplementary file 1 — Additional file 1. [file 12883_2021_2328_MOESM1_ESM.docx]

**Inner diameters of the normal** **carotid arteries measured using three-dimensional digital subtraction catheter angiography: a retrospective analysis**

Qingjing Tan^1^, Chao Qin ^2^, Junwei Yang^1^, Tianbao Wang^1^, Haohai Lin^1^, Cuiting Lin^2^, Xiangren Chen^2^

1. Department of Encephalopathy, First Affiliated Hospital of Guangxi University of Chinese Medicine, Nanning 530023, China.

2. Department of Neurology, First Affiliated Hospital of Guangxi Medical University, Nanning 530021, China.

Corresponding author: Chao Qin, E-mail: qc663902@126.com

**Supplemental Table 1. Calculation of Essen Stroke Risk Score (ESRS).**

| Risk factor | Score |
| --- | --- |
| Age < 65 years / 65–75 years / > 75 years | 0 / 1 / 2 |
| Hypertension | 1 |
| Diabetes mellitus | 1 |
| Previous myocardial infarction | 1 |
| Other heart diseases (excluding cardiac infarction and atrial fibrillation) | 1 |
| Smoker | 1 |
| Previous history of ischemic stroke/transient ischemic attack | 1 |

ESRS < 3 points was considered low-risk, ESRS ≥3 points was considered high-risk.
